# Supplementary material for: Fitness consequences of targeted gene flow to counter impacts of drying climates on terrestrial-breeding frogs
Source: Commun Biol. 2021 Oct 18;4:1195. doi: 10.1038/s42003-021-02695-w (PMC8523558; doi:10.1038/s42003-021-02695-w)
Supplement: Supplementary file 1 — Supplementary Information [file 42003_2021_2695_MOESM1_ESM.pdf]

Supplementary Information

Fitness consequences of targeted gene flow to counter impacts of drying climates on terrestrial-breeding frogs

Rudin-Bitterli, T.S., Evans, J.P. & Mitchell, N.J.

Supplementary Table 1. Results from a mixed-effects model of traits associated with desiccation tolerance in offspring originating from within- and between- crosses of four *P. guentheri* populations.

Trait	*N*	Source	df	X^2	*P*	Sig.
Embryonic survival (Proportion of fertilised eggs hatching)	2973	Treatment	1	20570	< 0.001	***
		Female origin	2	7.857	0.020	*
		Male origin	3	180.845	< 0.001	***
		Female origin x Male origin	6	214.388	< 0.001	***
		Female origin x Treatment	2	0.669	0.326	ns
		Male origin x Treatment	3	4.741	0.192	ns
		Female origin x Male origin x Treatment	6	3.283	0.773	ns
		Ovum size	1	2.823	0.093	ns
Time to hatching (h)	1751	Treatment	1	444.220	< 0.001	***
		Female origin	2	143.549	< 0.001	***
		Male origin	3	462.035	< 0.001	***
		Female origin x Male origin	6	38.296	< 0.001	***
		Female origin x Treatment	2	23.257	< 0.001	***
		Male origin x Treatment	3	49.722	< 0.001	***
		Female origin x Male origin x Treatment	6	17.555	0.007	**
		Ovum size	1	14.702	< 0.001	***
Wet weight at hatching (mg)	1746	Treatment	1	653.742	< 0.001	***
		Female origin	2	6.243	0.044	*
		Male origin	3	120.867	< 0.001	***
		Female origin x Male origin	6	23.817	< 0.001	***
		Female origin x Treatment	2	102.227	< 0.001	***
		Male origin x Treatment	3	2.417	0.491	ns
		Female origin x Male origin x Treatment	6	19.874	0.003	**
		Ovum size	1	17.985	< 0.001	***
Gosner Stage at hatching	1460	Treatment	1	113.773	< 0.001	***
		Female origin	2	292.710	< 0.001	***
		Male origin	3	605.253	< 0.001	***
		Female origin x Male origin	6	285.239	< 0.001	***
		Female origin x Treatment	2	38.723	< 0.001	***
		Male origin x Treatment	3	6.892	0.075	ns
		Female origin x Male origin x Treatment	6	21.090	0.002	**
		Ovum size	1	7.846	0.005	**

Supplementary Table 1 continued.

Trait	*N*	Source	df	X^2	*P*	Sig.
Proportion of malformed tadpoles	1751	Treatment	1	16.945	< 0.001	***
		Female origin	2	24.689	< 0.001	***
		Male origin	3	39.446	< 0.001	***
		Female origin x Male origin	6	40.540	< 0.001	***
		Female origin x Treatment	2	8.757	0.013	*
		Male origin x Treatment	3	1.341	0.719	ns
		Female origin x Male origin x Treatment	6	3.095	0.797	ns
		Ovum size	1	3.544	0.060	ns

Sig. = significance. Stronger statistical significance is indicated by more asterisks, and ns indicates no significance.

Supplementary Table 2. Results from a mixed-effects model of swimming performance traits in *P. guentheri* tadpoles originating from within- and between- crosses of four *P. guentheri* populations.

Trait	*N*	Source	df	X^2	*P*	Sig.
Maximum velocity (cm s^{-1})	633	Treatment	1	168.421	< 0.001	***
		Female origin	2	0.832	0.660	ns
		Male origin	3	98.328	< 0.001	***
		Female origin x Male origin	6	71.917	< 0.001	***
		Female origin x Treatment	2	21.469	< 0.001	***
		Male origin x Treatment	3	8.637	0.035	*
		Female origin x Male origin x Treatment	6	4.139	0.658	ns
		Ovum size	1	9.169	0.002	**
Mean velocity (cm s^{-1})	633	Treatment	1	59.307	< 0.001	***
		Female origin	2	48.836	< 0.001	***
		Male origin	3	48.201	< 0.001	***
		Female origin x Male origin	6	40.915	< 0.001	***
		Female origin x Treatment	2	15.138	< 0.001	***
		Male origin x Treatment	3	3.384	0.336	ns
		Female origin x Male origin x Treatment	6	7.177	0.305	ns
		Ovum size	1	3.459	0.063	ns
Mean meander (deg cm^{-1})	633	Treatment	1	3.593	0.058	ns
		Female origin	2	105.812	< 0.001	***
		Male origin	3	39.759	< 0.001	***
		Female origin x Male origin	6	30.406	< 0.001	***
		Female origin x Treatment	2	8.129	0.017	*
		Male origin x Treatment	3	1.839	0.607	ns
		Female origin x Male origin x Treatment	6	15.130	0.019	*
		Ovum size	1	1.688	0.194	ns
Total distance moved (cm)	633	Treatment	1	6.917	0.008	**
		Female origin	2	5.014	0.082	ns
		Male origin	3	1.003	0.801	ns
		Female origin x Male origin	6	14.762	0.022	*
		Female origin x Treatment	2	3.503	0.174	ns
		Male origin x Treatment	3	10.486	0.015	*
		Female origin x Male origin x Treatment	6	5.509	0.480	ns
		Ovum size	1	0.238	0.625	ns

Sig. = significance. Stronger statistical significance is indicated by more asterisks, and ns indicates no significance.
