# Supplementary material for: Fitness consequences of targeted gene flow to counter impacts of drying climates on terrestrial-breeding frogs
Source: Commun Biol. 2021 Oct 18;4:1195. doi: 10.1038/s42003-021-02695-w (PMC8523558; doi:10.1038/s42003-021-02695-w)
Supplement: Supplementary file 3 — Reporting Summary [file 42003_2021_2695_MOESM3_ESM.pdf]

nature research

Corresponding author(s): COMMSBIO-21-0822-T
Nicola Mitchell

Last updated by author(s): 03/08/2021

Reporting Summary

Nature Research wishes to improve the reproducibility of the work that we publish. This form provides structure for consistency and transparency in reporting. For further information on Nature Research policies, see our Editorial Policies and the Editorial Policy Checklist.

Statistics

For all statistical analyses, confirm that the following items are present in the figure legend, table legend, main text, or Methods section.

n/a	Confirmed	
☐	☒	The exact sample size (*n*) for each experimental group/condition, given as a discrete number and unit of measurement
☐	☒	A statement on whether measurements were taken from distinct samples or whether the same sample was measured repeatedly
☐	☒	The statistical test(s) used AND whether they are one- or two-sided<br>*Only common tests should be described solely by name; describe more complex techniques in the Methods section.*
☐	☒	A description of all covariates tested
☐	☒	A description of any assumptions or corrections, such as tests of normality and adjustment for multiple comparisons
☐	☒	A full description of the statistical parameters including central tendency (e.g. means) or other basic estimates (e.g. regression coefficient) AND variation (e.g. standard deviation) or associated estimates of uncertainty (e.g. confidence intervals)
☐	☒	For null hypothesis testing, the test statistic (e.g. F, t, r) with confidence intervals, effect sizes, degrees of freedom and P value noted<br>*Give P values as exact values whenever suitable.*
☒	☐	For Bayesian analysis, information on the choice of priors and Markov chain Monte Carlo settings
☒	☐	For hierarchical and complex designs, identification of the appropriate level for tests and full reporting of outcomes
☒	☐	Estimates of effect sizes (e.g. Cohen's d, Pearson's r), indicating how they were calculated

Our web collection on statistics for biologists contains articles on many of the points above.

Software and code

Policy information about availability of computer code

Data collection	We used EthoVision v8.5 software to analyse the burst swimming responses of hatchlings.
Data analysis	All analyses were performed in R version 3.4.3

For manuscripts utilizing custom algorithms or software that are central to the research but not yet described in published literature, software must be made available to editors and reviewers. We strongly encourage code deposition in a community repository (e.g. GitHub). See the Nature Research guidelines for submitting code & software for further information.

Data

Policy information about availability of data

All manuscripts must include a data availability statement. This statement should provide the following information, where applicable:

- Accession codes, unique identifiers, or web links for publicly available datasets
- A list of figures that have associated raw data
- A description of any restrictions on data availability

Data and R scripts supporting the findings of this study are available in Dryad with the identifier doi:10.5061/dryad.6m905qg09

Field-specific reporting

Please select the one below that is the best fit for your research. If you are not sure, read the appropriate sections before making your selection.

☐ Life sciences ☐ Behavioural & social sciences ☒ Ecological, evolutionary & environmental sciences

For a reference copy of the document with all sections, see nature.com/documents/nr-reporting-summary-flat.pdf

Ecological, evolutionary & environmental sciences study design

All studies must disclose on these points even when the disclosure is negative.

Study description	We created mixed- and within-population crosses in a terrestrial-breeding frog species threatened by a drying climate to assess the potential of targeted gene flow to increase the adaptive potential of these populations to a drying climate. We reared embryos on wet and dry soils and quantified fitness-related traits upon hatching. Linear mixed-effects models (with restricted maximum-likelihood methods; REML) were run to compare offspring traits from pure and hybrid crosses. In these models, treatment, female (population) origin, male (population) origin and all interactions (female origin-by-male origin, female origin-by-treatment, male origin-by-treatment, female origin-by-male origin-by-treatment) were considered as fixed factors. Individual female ID was added as a random effect and ovum size was added as covariate to all models.
Research sample	Pseudophryne guentheri adults (males and females) from 4 populations in Western Australia, and F1 offspring from crosses thereof (within and between-population).
Sampling strategy	Sampling size was informed by the successful sampling strategy of a previous study which assessed the geographic variation in desiccation tolerance in the same frog populations across a rainfall gradient.
Data collection	The data collection procedure is described in the methods section of the manuscript. We did not perform any other data collection that is not described within the methods section. Data was mainly collected by Tabitha Rudin-Bitterli, with assistance from staff and volunteers who helped with the collection of adults in the field and measuring response variables of hatchlings in the laboratory. These people were Brighton Downing, Deanne Cummins, Blair Bentley, Jon-Paul Emery and Emily Hoffman. Nicola Mitchell provided training to Tabitha in some relevant laboratory procedures, such as in-vitro fertilisation methods and euthanasia of adult animals.
Timing and spatial scale	Adults were collected in May and June 2017, and laboratory crosses were performed shortly after collection of adults. Hatching of F1 offspring was induced 33 days after fertilisation.
Data exclusions	No data were excluded.
Reproducibility	All attempts to repeat the experiment (via increasing sample size) were successful. This study builds on the results of a previous study which assessed the geographic variation in desiccation tolerance in the same frog populations across a rainfall gradient. This previous study performed within-population crosses of these populations only, and assessed the same response parameters using the same techniques as the present manuscript. The results from the within-population crosses from the present manuscript are comparable to the results of the previous study, suggesting that these results are reproducible.
Randomization	We used randomisation wherever possible. A sperm sample from five random males was used in fertilisations, fertilised eggs from each cross were selected at random and distributed onto soils at two water potentials. Swimming performance was assessed on a subset of hatchlings, for which specimens from each cross were selected randomly.
Blinding	Where possible, we used software to measure response variables, such as the use of EthoVision to measure the swimming responses of hatchlings to remove any potential observer bias. Otherwise, blinding was quite difficult to implement during this study, as hatchlings from dry and wet treatments were easily recognisable (even with blinding) due to their morphological differences.

Did the study involve field work? ☒ Yes ☐ No

Field work, collection and transport

Field conditions	Conditions in the field were usually wet (during and/or after rainfall), dark (as sampling was conducted at night), and temperatures ranged between approximately 10 and 20 degrees Celsius
Location	Chidlow: 31°53'05.5"S, 116°18'48.0"E<br>Ridgefield: 32°28'25.9"S, 116°58'27.9"E<br>Binnu: 28°02'30.8"S, 114°39'36.0"E<br>Mullewa: 28°31'07.3"S, 115°38'11.4"E
Access & import/export	All animal procedures were approved by the University of Western Australia's (UWA) Animal Ethics Committee (permit number RA/3/100/1510), and the research was conducted under animal collection license 08-000560-1 from the Western Australian Department of Biodiversity, Conservation and Attractions. Permission from all landholders was sought before conducting any field work.
Disturbance	The main disturbances of the study were connected to the pit-fall trapping of adult Pseudophryne guentheri individuals. Where possible, individuals were collected by hand. Nevertheless, as females in particular are difficult to collect, some pit-fall trapping had

to be conducted. We worked closely with the UWA ethics committee to minimise any disturbances to the animals. Pit-fall traps were frequently checked throughout the night and always closed during the day. Shelter was provided in each pit-fall trap, including protection from abrupt temperature changes via Styrofoam cups. Pit-fall traps were removed immediately after sufficient animals had been caught. The study recorded no mortalities associated with the collection of animals from the field.

Reporting for specific materials, systems and methods

We require information from authors about some types of materials, experimental systems and methods used in many studies. Here, indicate whether each material, system or method listed is relevant to your study. If you are not sure if a list item applies to your research, read the appropriate section before selecting a response.

Materials & experimental systems

n/a	Involved in the study	
☐	☐	Antibodies
☐	☐	Eukaryotic cell lines
☐	☐	Palaeontology and archaeology
☐	☒	Animals and other organisms
☐	☐	Human research participants
☐	☐	Clinical data
☐	☐	Dual use research of concern

Methods

n/a	Involved in the study	
☐	☐	ChIP-seq
☐	☐	Flow cytometry
☐	☐	MRI-based neuroimaging

Antibodies

Antibodies used	no antibodies were used
Validation	NA

Eukaryotic cell lines

Policy information about cell lines

Cell line source(s)	no cell lines were used
Authentication	NA
Mycoplasma contamination	NA
Commonly misidentified lines (See ICLAC register)	NA

Palaeontology and Archaeology

Specimen provenance	NA
Specimen deposition	NA
Dating methods	NA

☐ Tick this box to confirm that the raw and calibrated dates are available in the paper or in Supplementary Information.

Ethics oversight	NA

Note that full information on the approval of the study protocol must also be provided in the manuscript.

Animals and other organisms

Policy information about studies involving animals; ARRIVE guidelines recommended for reporting animal research

Laboratory animals	The study did not involve laboratory animals.
Wild animals	We collected 61 adult male Pseudophryne guentheri, and 24 gravid females from 4 populations in WA. Frogs were either collected by hand (preferred) or via pit-fall traps. After collection, frogs were temporarily housed in small (4.4 L) plastic terraria containing moist sphagnum moss and pinhead crickets, and transported to the University of Western Australia within two days of collection. There, frogs were fed a diet of pinhead crickets and kept in a controlled-temperature room at 16 °C with an 11/13 h light/dark photoperiod to mimic winter conditions. All animals were euthanised after the experiments. Males had to be euthanised to collect the sperm (non-invasive methods that produce large amounts of viable sperm are not available at present). We worked with the Western Australian Department of Biodiversity, Conservation and Attractions, who oversees animal collections in Western Australia, to

	determine the fate of female frogs collected during our study. Ultimately, they decided that the risks (i.e. introduction of diseases etc.) of reintroducing these females to their collection site outweighed the benefits, and females were euthanised immediately after in-vitro fertilisations. All animals were euthanised via ventral immersion in <0.03% benzocaine solution, followed by double pithing.
Field-collected samples	The study did not involve samples collected from the field.
Ethics oversight	All animal procedures were approved by the University of Western Australia's (UWA) Animal Ethics Committee (permit number RA/3/100/1510), and the research was conducted under animal collection license 08-000560-1 from the Western Australian Department of Biodiversity, Conservation and Attractions.

Note that full information on the approval of the study protocol must also be provided in the manuscript.

Human research participants

Policy information about studies involving human research participants

Population characteristics	NA
Recruitment	NA
Ethics oversight	NA

Note that full information on the approval of the study protocol must also be provided in the manuscript.

Clinical data

Policy information about clinical studies

All manuscripts should comply with the ICMJE guidelines for publication of clinical research and a completed CONSORT checklist must be included with all submissions.

Clinical trial registration	NA
Study protocol	NA
Data collection	NA
Outcomes	NA

Dual use research of concern

Policy information about dual use research of concern

Hazards

Could the accidental, deliberate or reckless misuse of agents or technologies generated in the work, or the application of information presented in the manuscript, pose a threat to:

No	Yes	
☒	☐	Public health
☒	☐	National security
☒	☐	Crops and/or livestock
☒	☐	Ecosystems
☒	☐	Any other significant area

Experiments of concern

Does the work involve any of these experiments of concern:

No	Yes	
☒	☐	Demonstrate how to render a vaccine ineffective
☒	☐	Confer resistance to therapeutically useful antibiotics or antiviral agents
☒	☐	Enhance the virulence of a pathogen or render a nonpathogen virulent
☒	☐	Increase transmissibility of a pathogen
☒	☐	Alter the host range of a pathogen
☒	☐	Enable evasion of diagnostic/detection modalities
☒	☐	Enable the weaponization of a biological agent or toxin
☒	☐	Any other potentially harmful combination of experiments and agents

ChIP-seq

Data deposition

☐ Confirm that both raw and final processed data have been deposited in a public database such as GEO.

☐ Confirm that you have deposited or provided access to graph files (e.g. BED files) for the called peaks.

Data access links<br>*May remain private before publication.*	NA
Files in database submission	NA
Genome browser session<br>(e.g. UCSC)	NA

Methodology

Replicates	NA
Sequencing depth	NA
Antibodies	NA
Peak calling parameters	NA
Data quality	NA
Software	NA

Flow Cytometry

Plots

Confirm that:

☐ The axis labels state the marker and fluorochrome used (e.g. CD4-FITC).

☐ The axis scales are clearly visible. Include numbers along axes only for bottom left plot of group (a 'group' is an analysis of identical markers).

☐ All plots are contour plots with outliers or pseudocolor plots.

☐ A numerical value for number of cells or percentage (with statistics) is provided.

Methodology

Sample preparation	NA
Instrument	NA
Software	NA
Cell population abundance	NA
Gating strategy	NA

☐ Tick this box to confirm that a figure exemplifying the gating strategy is provided in the Supplementary Information.

Magnetic resonance imaging

Experimental design

Design type	NA
Design specifications	NA
Behavioral performance measures	NA

Acquisition

Imaging type(s)	NA
Field strength	NA
Sequence & imaging parameters	NA
Area of acquisition	NA

Diffusion MRI ☐ Used ☒ Not used

Preprocessing

Preprocessing software	NA
Normalization	NA
Normalization template	NA
Noise and artifact removal	NA
Volume censoring	NA

Statistical modeling & inference

Model type and settings	NA
Effect(s) tested	NA

Specify type of analysis: ☐ Whole brain ☐ ROI-based ☐ Both

Statistic type for inference (See Eklund et al. 2016)	NA
Correction	NA

Models & analysis

n/a	Involved in the study
☐	☐ Functional and/or effective connectivity
☐	☐ Graph analysis
☐	☐ Multivariate modeling or predictive analysis

Functional and/or effective connectivity	NA
Graph analysis	NA
Multivariate modeling and predictive analysis	NA
